# NGAL and traditional biomarkers in cervical cancer: a comparative evaluation using random forest

**DOI:** 10.3389/fonc.2026.1933456

**Published:** 2026-10-20

**Authors:** Behar Raci, Snezana Stojkovska, Gezim Hodolli, Violeta Klisarovska, Goran Dimitrov, Arta Kameri Jusufi, Hekuran Sejdiu, Burim Sermaxhaj, Diellor Rizaj, Ilir Kurtishi, Mentor Kurshumliu

**Affiliations:** 1 Clinic of Oncology, University Clinical Center of Kosovo, Prishtina, Kosovo; 2 University Clinic for Infectious Diseases & Febrile Conditions, Faculty of Medicine, Skopje, North Macedonia; 3 University Clinic of Radiotherapy and Oncology, Faculty of Medicine, Skopje, North Macedonia; 4 University Clinic of Gynecology and Obstetrics, Faculty of Medicine, Skopje, North Macedonia; 5 Clinic for Radio-Oncology, Stadtspital Triemli, Zyrich, Switzerland; 6 UBT College, Prishtine, Kosovo; 7 College of Medical Sciences Heimerer, Prishtina, Kosovo

**Keywords:** biomarkers, cervical cancer, NGAL, random forest, vitamin D

## Abstract

**Introduction:**

Tumor stage and biological aggressiveness have a significant impact on the prognosis of cervical cancer, which is still a serious global health concern.

**Methods:**

This work assesses an exploratory, leakage-free Random Forest model and describes longitudinal NGAL and traditional biomarker patterns in 100 individuals with cervical cancer. Since there was no independent radiologic, pathological, or defined response endpoint in the database, the machine-learning result was specifically characterized as an NGAL-decrease phenotype (post-treatment NGAL < pre-treatment NGAL), not clinical treatment response. All NGAL-derived variables and all post-treatment variables were removed from the predictor set in order to prevent target leakage.

**Results:**

The mean accuracy using stratified 5-fold cross validation was 43.0% (SD 7.6%) and the mean balanced accuracy was 42.1%, suggesting that there was no discriminative performance that was useful. Greater histological grades and more advanced broad-stage groups had descriptively greater NGAL concentrations.

**Discussion:**

These results do not prove clinical predictive usefulness; rather, they are hypothesis-generating.

## 1 Introduction

Cervical cancer remains one of the most prevalent malignancies among women worldwide, particularly in low- and middle-income countries. Despite advances in screening and vaccination programs, a significant number of patients are still diagnosed at advanced stages, requiring aggressive treatment modalities such as chemoradiotherapy (1). The ability to predict individual patient response to treatment remains a major challenge in gynecologic oncology.

Recent years have seen increased interest in the role of biomarkers that reflect systemic inflammation, tumor burden, and metabolic status as predictive tools in cancer therapy. Among these, neutrophil gelatinase-associated lipocalin (NGAL) has emerged as a biomarker of interest due to its involvement in inflammation, immune response, and tumor progression (2, 3). NGAL is a protein that is secreted by activated neutrophils (3). Although neutrophils are the main source of NGAL, their expression is also found in numerous human tissues, including tubular cells in the kidney, heart, lung, liver, stomach, colon, epithelial cells, macrophages, dendritic cells and adipocytes (4–10). NGAL is also known as lipocalin-2 and represents a family of lipocalin proteins (11). Its structure is often described as a three-dimensional barrel (12).

NGAL plays a role as an acute phase protein and takes part in antibacterial immune processes. Inflammatory cytokines induce NGAL expression in neutrophils, epithelial cells, or hepatocytes (13–15).

NGAL levels have been shown to correlate with disease severity and prognosis in several cancers, including cervical cancer, and may serve as a real-time marker of treatment effectiveness (16). In general, high concentrations of NGAL have been associated with advanced cancer and poor prognosis (17).

In parallel, computational methods such as machine learning have gained ground in the analysis of complex clinical and biological data. Random Forest algorithms, in particular, offer advantages including robustness to overfitting, handling of high-dimensional data, and interpretability through feature importance metrics (18). These characteristics make Random Forests highly suitable for modeling multifactorial conditions such as cancer response to therapy. For example, a very recent study applied an advanced ensemble model bagging combined with histogram gradient boosting and optimized feature selection to predict the onset of cardiovascular diseases, achieving excellent performance and outperforming classical machine-learning methods on multiple datasets (19). Furthermore, cutting-edge evaluations in the field of gynecological oncology suggest that integrating deep-learning or ML-based algorithms with molecular biomarkers can greatly improve diagnostic sensitivity, specificity, and prognosis accuracy in cervical cancer detection and classification (20).

The mechanisms underlying the way NGAL promotes human cancer has been explored in several studies (2), highlighting how NGAL furthers iron uptake from the extracellular space within the malignant cells to maintain multiplication of neoplastic cells (D).

At the national level, there is a lack of studies of this kind. Although a few investigations have been conducted so far (9), this study aims to contribute to disease prevention and prognosis by utilizing NGAL as a biomarker.

In order to determine if baseline, non-NGAL characteristics may use Random Forest to define a subsequent NGAL-decrease phenotype, this study sought to characterize pre- and post-treatment patterns of NGAL and conventional circulating biomarkers. The machine-learning component is explicitly exploratory and is not offered as a forecast of radiologic, pathological, or clinical treatment response because no independent objective clinical-response outcome was recorded in the provided database. Operability, broad FIGO stage, and histological grade subgroup analyzes were exploratory rather than predetermined confirmatory analyzes.

## 2 Materials and methods

One hundred female patients treated at the Oncology Clinic of the University Clinical Center of Kosovo (UCCK), Pristina, for histologically proven cervical cancer were included in this study. In accordance with institutional practice, the course of treatment was modified. 2 × 9 Gy (18 Gy) was administered to a subset of early-stage brachytherapy patients. In addition to brachytherapy (2 × 9 Gy), patients with non-metastatic locally progressed disease were treated with IMRT/VMAT to 50.4 Gy in 28 fractions (1.8 Gy/fraction), followed where warranted by a 5.4 Gy boost in 3 fractions. When clinically suitable, concurrent cisplatin was given once a week at a dose of 50 mg/m² for a maximum of five cycles. The treatment modality is recorded in the database, but the precise number of cisplatin cycles each patient completes is not. Written informed consent was given by each subject. The Kosovo Doctors’ Committee gave its ethical approval. Written informed consent was given by each subject. The Kosovo Doctors’ Chamber’s Commission for Ethical Affairs gave its ethical approval (Ref. No. 124/23, July 2023). Sampling and recruitment was place between July 2023 and July 2024.

The inclusion criteria required female participants aged 25–75 years with a histologically confirmed diagnosis of cervical cancer. In addition to the patient group, a control group of 30 healthy women aged 20 years or older was included. Control participants voluntarily agreed to participate by signing informed consent forms and had no personal or family history of cancer, chronic illness, or ongoing pathological conditions.

The exclusion criteria ruled out patients with systemic or chronic diseases that could influence biochemical or hematological outcomes. Patients with diabetes mellitus, autoimmune disorders, hypertension, hyperlipidemia, or viral infections were excluded. Those with impaired renal function—defined as urea or creatinine levels exceedingly twice the upper reference limit—or hepatic impairment, characterized by AST, ALT, total bilirubin, or direct bilirubin values more than twofold above reference levels, were also excluded to avoid confounding effects.

There were 100 patients in the final dataset that could be analyzed (mean age 55.9 years; range 29–75). Of the patients, 38 were inoperable and 62 were operable. Eighty squamous/planocellular carcinomas, eighteen adenocarcinomas, and two microinvasive carcinomas were found in the histology. Nine individuals had a histological grade of G1, 68 had a grade of G2, and 23 had a grade of G3. The illness stage is conservatively summarized by broad FIGO group: stage I, n = 45; stage II, n = 23; stage III, n = 30; and 2 records with inadequate standardized stage information in order to prevent assigning non-standard local suffixes to FIGO 2018 substages without appropriate nodal-location information. 67 patients received CCRT with brachytherapy, 13 received RT plus brachytherapy, 12 had brachytherapy alone, 6 received CCRT without a documented brachytherapy entrance, and 2 received no treatment.

NGAL, vitamin D, CEA, CA-125, WBC, RBC, HGB, HCT, PLT, ionized calcium, NLR, and MLR were among the laboratory variables for each participant. The NGAL modification itself was used to define the initial binary variable, “response.” It is now known as the “NGAL-decrease phenotype” in the updated analysis, which defines it as post-treatment NGAL < pre-treatment NGAL. This outcome is not comparable to objective clinical, radiologic, or pathological response; rather, it is a biomarker-derived phenotype. Of the patients, 53 satisfied the NGAL-decrease criteria, while 47 did not.

Blood samples were taken at two predetermined intervals: three to five days before to the start of EBRT/chemoradiotherapy (or roughly three days prior to brachytherapy in patients in the early stages) and two to four weeks following the conclusion of treatment. Throughout this revision, NGAL specimen terminology has been standardized to EDTA plasma based on the published K_2_EDTA collection process. The Human NGAL ELISA Kit (KIT 036CE, BioPorto Diagnostics, Hellerup, Denmark) was used to measure NGAL. A Sysmex KX-21N analyzer was used to measure hematologic parameters in whole blood. The Architect i1000 SR system was used to measure vitamin D. These details are not inferred here and should be verified from the laboratory SOP/kit documentation prior to final submission because the provided patient database lacks assay-platform metadata for CEA and CA-125 as well as analytical performance fields like intra-/inter-assay coefficients of variation and replicate status.

statistical evaluation. When presented descriptively, continuous variables were summed up using mean and standard deviation. Because group sizes varied, Welch’s two-sample t-test was utilized to assess the pre-treatment NGAL comparison between the operable and inoperable groups; a paired t-test was employed for the pre/post NGAL comparison. Exploratory summaries were separated by grade and stage. To remove direct target leakage, a Random Forest classifier (MATLAB analysis theoretically recreated in the provided database revision) was re-specified for machine learning. Age, grade, WBC, RBC, HGB, HCT, PLT, vitamin D, CEA, CA-125, ionized calcium, NLR, MLR, operability, and broad FIGO stage were the only pre-treatment, non-NGAL characteristics that could be used as predictors. Every post-treatment variable and every NGAL variable were eliminated. Class balancing was used, categorical variables were encoded, and missing numerical values were median-imputed. Using stratified 5-fold cross-validation with a fixed random seed, a 1000-tree Random Forest with a minimum leaf size of 2 was assessed (42). There were reports on accuracy, balanced accuracy, precision, recall, F1-score, confusion matrix, and Mean Decrease in Gini feature relevance. The analysis lacked specifications for confirmatory inference and was exploratory in nature.

Therefore, rather than predicting clinical treatment response, the Random Forest analysis was intended to determine whether baseline non-NGAL information could classify the following NGAL-decrease phenotype. There is no claim of clinical predictive validity because the database does not have an independent objective response endpoint.

Mean was used to quantify the relevance of each feature. Gini impurity decreases for descriptive ranking only when the leakage-free model is fitted to the entire dataset. Feature relevance does not determine independent prognostic or predictive value; rather, it characterizes internal model splitting behavior.

## 3 Results

The NGAL-decrease phenotype was categorized using the leakage-free exploratory Random Forest utilizing only baseline non-NGAL predictors. The mean accuracy was 43.0% (SD 7.6%; fold range 35.0–55.0%) and the mean balanced accuracy was 42.1% (SD 8.1%) over stratified 5-fold cross-validation. 43 out of 100 patients were properly classified using cross-validated predictions. These results show no discrimination that is useful.

NGAL_pre, NGAL_post, NGAL_delta, and any post-treatment variable are no longer included in the corrected model’s feature-importance ranking. WBC (0.099), HCT (0.089), vitamin D (0.087), age (0.082), NLR (0.082), PLT (0.079), HGB (0.077), CA125 (0.073), ionized calcium (0.070), RBC (0.069), MLR (0.065), and CEA (0.063) were the top characteristics. The only reason these rankings are descriptive is that overall discrimination was subpar.

The leakage-free Random Forest feature-importance ranking is shown in **Figure 1**. Relative ranking should not be used as proof of clinical predictive value in the absence of robust classification performance.

**Figure 1:**
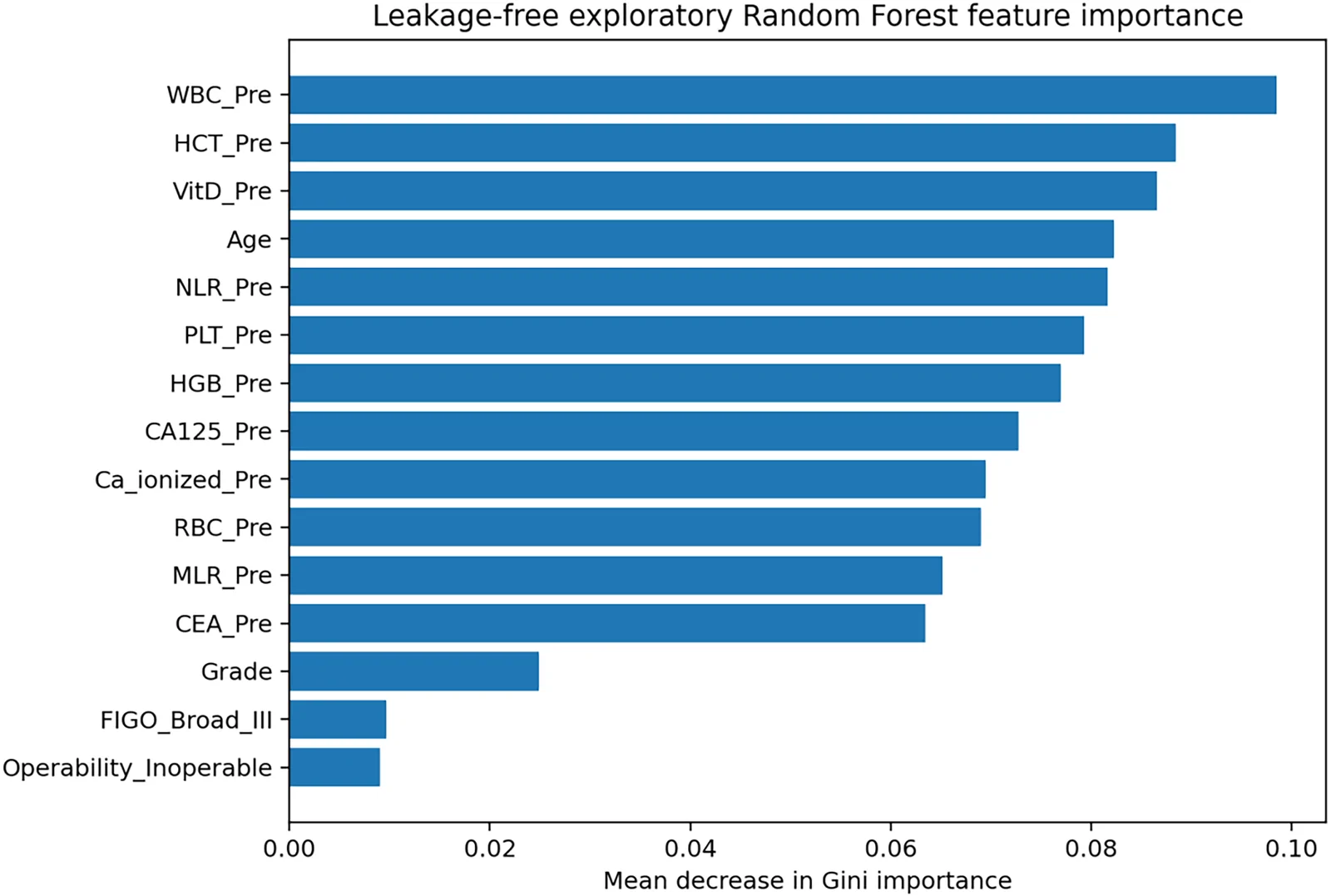
Leakage-free exploratory Random Forest feature importance using pre-treatment non-NGAL predictors only.

True class 0 (no NGAL reduction) had 14 correct classifications and 33 misclassifications in the cross-validated confusion matrix; true class 1 (NGAL decline) had 29 right classifications and 24 misclassifications. Overall accuracy was 43.0%.

Class 0 had precision of 0.37, recall of 0.30, and F1-score of 0.33; class 1 had precision of 0.47, recall of 0.55, and F1-score of 0.50. The macro-average F1 was 0.42.

Therefore, the previously reported 70.59% holdout accuracy and 56% mean cross-validation estimate from the leakage-prone model are replaced by the updated cross-validation findings. In the updated manuscript, those previous values are no longer used as performance indicators.

The cross-validated confusion matrix for each of the 100 patients using the modified leakage-free model is shown in **Table 2**.

**Table 1:** Baseline clinical characteristics of the 100-patient cohort.

Category	Predicted no decrease	Predicted NGAL decrease
True No decrease	14	33
True NGAL decrease	24	29
Inoperable	38 (38%)	
Histology: squamous/planocellular	80 (80%)	
Histology: adenocarcinoma	18 (18%)	
Histology: microinvasive carcinoma	2 (2%)	
Grade 1	9 (9%)	
Grade 2	68 (68%)	
Grade 3	23 (23%)	
Broad FIGO stage I	45 (45%)	
Broad FIGO stage II	23 (23%)	
Broad FIGO stage III	30 (30%)	
Stage uncertain/not standardized	2 (2%)	
CCRT + brachytherapy	67 (67%)	
RT + brachytherapy	13 (13%)	
Brachytherapy alone	12 (12%)	
CCRT	6 (6%)	
Treatment not recorded	2 (2%)	

**Table 2:** Cross-validated confusion matrix for the leakage-free random forest model.

Class	Precision	Recall	F1-score	Support
0: No decrease	0.37	0.30	0.33	47
1: NGAL decrease	0.47	0.55	0.50	53
Accuracy	/	/	0.43	100
Macro average	0.42	0.42	0.42	100
Weighted average	0.42	0.43	0.42	100

The associated cross-validated categorization metrics are shown in **Table 3**. Only the NGAL-decrease phenotypic classification is described in both tables; objective clinical treatment response is not.

**Table 3:** Cross-validated classification report for the leakage-free random forest model.

Label	Precision	Recall	F1-score	Support
0	0.44	0.5	0.47	8.0
1	0.5	0.44	0.47	9.0
accuracy	0.47	0.47	0.47	0.47
macro avg	0.47	0.47	0.47	17.0
weighted avg	0.47	0.47	0.47	17.0

No assertion of a dependable predictive structure or clinical decision-support value is made due to the poor performance of the revised model.

The current model cannot be said to have a credible predictive structure due to the heterogeneity among folds and the circular target specification. Predictive interpretation requires a leakage-free model with an independently determined clinical endpoint and external validation.

### 3.1 Comparative subgroup analysis based on operability

All 100 patients had operability available; 38 were deemed inoperable and 62 were deemed operable. The operable group’s mean pre-treatment NGAL was 123.49 ± 71.38 ng/mL, while the inoperable group’s was 156.22 ± 70.26 ng/mL (Welch’s t-test, p=0.027). This was an exploratory subgroup analysis, more detailed data can be found at **Table 1**.

Superior performance in operable versus inoperable subgroups was not claimed using the revised Random Forest. Due to the leakage-prone target/predictor design and few subgroup samples, previous subgroup model accuracies were eliminated.

The leakage-free exploratory model maintains operability as a baseline categorical predictor and as a descriptive clinical stratification variable.

There was no predetermined inferential comparison of subgroup-specific machine learning performance.

Although the subgroup machine-learning results are simply hypothesis-generating, operability stratification is nevertheless helpful for descriptive comparison of biomarker distributions. Instead of being predetermined confirmatory analyzes, these subgroup analyzes were exploratory.

### 3.2 NGAL by FIGO stage and histological grade

The updated analysis reports wide FIGO groups I, II, and III to prevent the spread of non-standard local staging suffixes that cannot be clearly translated to FIGO 2018 IIIC1/IIIC2 without pelvic versus para-aortic nodal-location data. Prior to any subsequent substage-level analysis, individual records that need IIIC1/IIIC2 assignment should be checked against source imaging and pathology.

Descriptively, pre-treatment NGAL was 103.9 ± 38.5 ng/mL in wide stage I (n = 45), 168.9 ± 71.6 ng/mL in stage II (n = 23), and 159.3 ± 91.9 ng/mL in stage III (n = 30). These comparisons are experimental and descriptive.

For broad stages I, II, and III, the post-treatment NGAL means were 98.4, 175.6, and 179.3 ng/mL, respectively. These alterations in the results are not explained mechanistically (e.g., nodal sterilization, therapy-induced inflammation, or residual illness).

Box-and-scatter plots with individual observations for NGAL before and after treatment across broad FIGO stages are shown in **Figure 2**. Because there is insufficient nodal-location metadata in the database to accurately recode every non-standard “c” suffix into FIGO 2018 IIIC1 versus IIIC2, the broad-stage technique is purposefully cautious.

**Figure 2:**
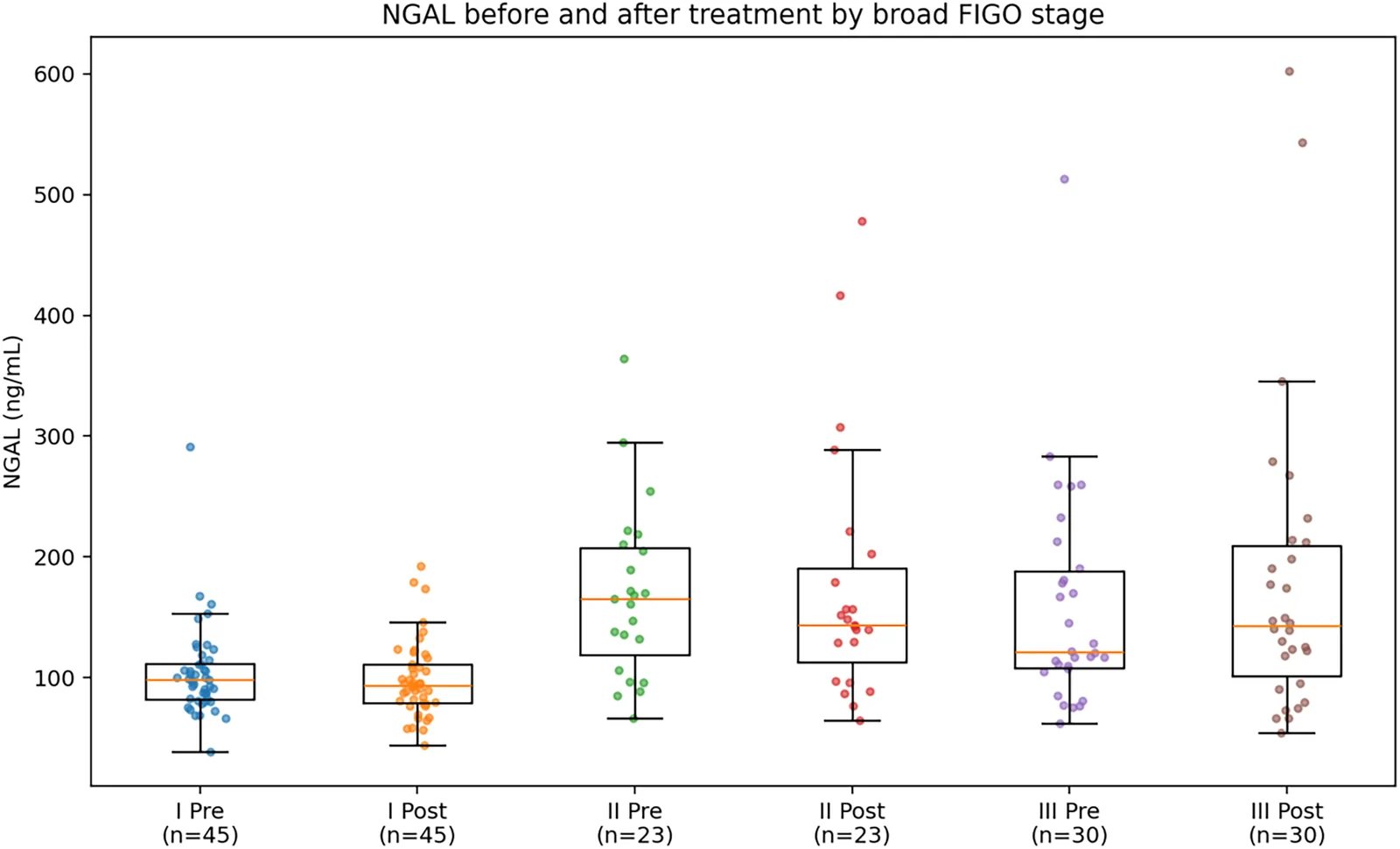
Pre- and post-treatment plasma NGAL distributions by broad FIGO stage. Individual points and boxplots are shown; n is indicated for each stage.

The mean pre-treatment NGAL by histological grade was 93.6 ± 40.5 ng/mL for G1 (n = 9), 125.1 ± 55.2 ng/mL for G2 (n = 68), and 184.6 ± 100.6 ng/mL for G3 (n = 23). The individual-data distributions prior to and following treatment are shown in **Figure 3**.

**Figure 3:**
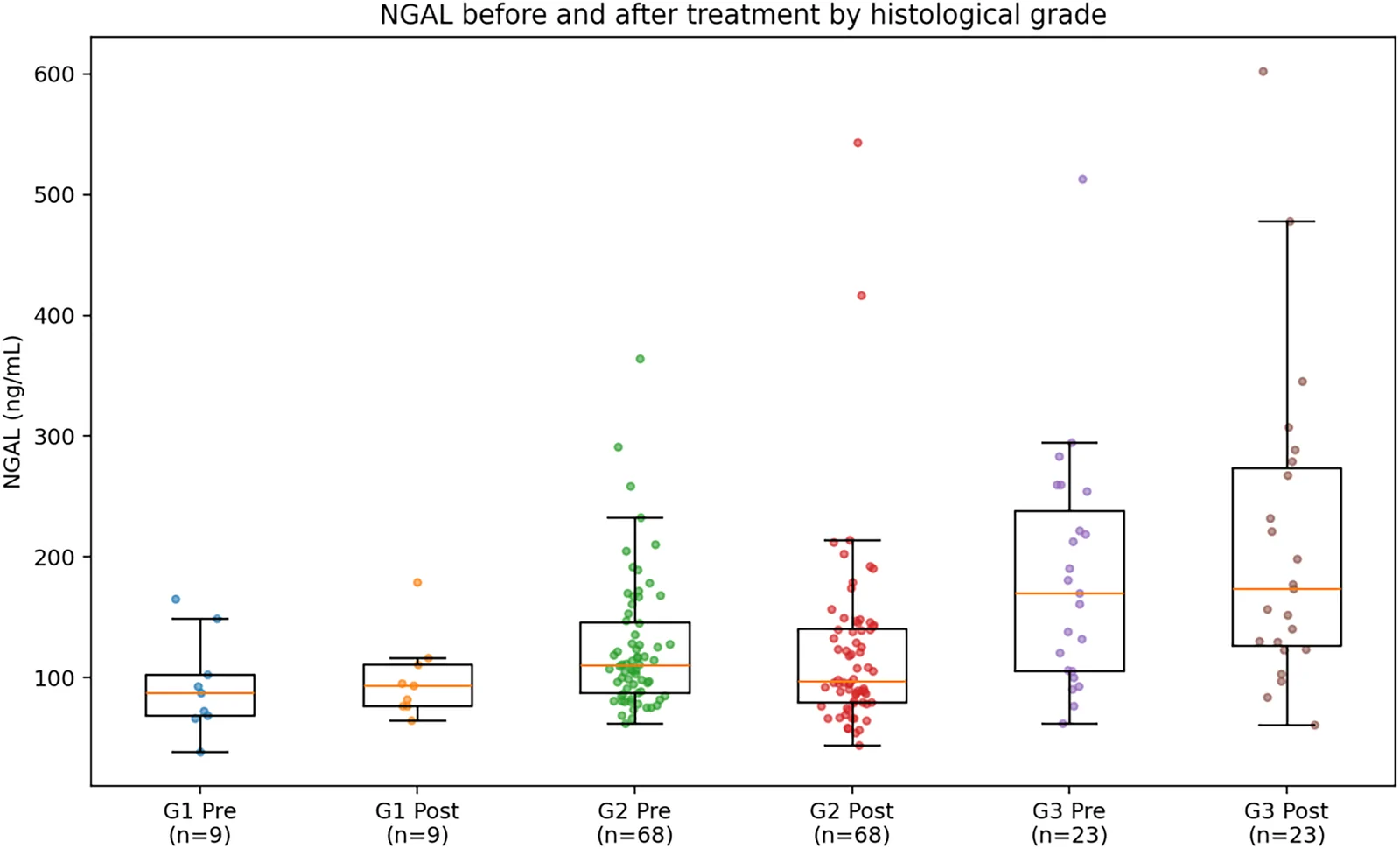
Pre- and post-treatment plasma NGAL distributions by histological grade. Individual points and boxplots are shown; n is indicated for each grade.

The grade-associated descriptive pattern in the Results does not suggest a biological mechanism; such processes are only discussed in the Discussion.

This investigation did not yield any clinically recognized NGAL cutoff, percentage-change threshold, imaging trigger, or treatment-adjustment rule. Due to the lack of a predetermined cutoff-validation analysis against an independent clinical objective, previously stated thresholds and clinical recommendations have been eliminated.

The current investigation could not provide a clinically validated NGAL cutoff for defining treatment response or differentiating between early and late cervical cancer. Because the manuscript does not report a prespecified ROC analysis, cutoff-selection method, sensitivity, specificity, confidence intervals, or independent clinical validation, the previously stated thresholds (>120 ng/mL and ≥15% post-treatment reduction) and recommendations regarding imaging or treatment adjustment have been removed.

## 4 Discussion

The current investigation could not provide a clinically validated NGAL threshold for identifying treatment response or differentiating between early and late cervical cancer. Since the manuscript does not report a prespecified ROC analysis, cutoff-selection method, sensitivity, specificity, confidence intervals, or independent clinical validation, the previously stated thresholds (>120 ng/mL and ≥15% post-treatment reduction) and recommendations regarding imaging or treatment adjustment have been eliminated.

NGAL and a number of traditional biomarkers were among the highly ranked variables in the initial feature-importance analysis. However, because the target label was derived from NGAL change, the prominence of NGAL_pre and NGAL_post cannot be taken as independent predictive evidence. Therefore, before clinical prognostic conclusions can be made, the machine-learning component needs a leakage-free re-analysis with an outcome specified independently of NGAL.

The comparable importance of Vitamin D pre- and post-treatment points to its relevance in modulating immune response and cancer progression. Meanwhile, fluctuations in platelet count (PLT) and calcium metabolism may reflect systemic stress and tissue remodeling processes induced by therapy.

The macro-average F1 was almost 0.42, and the cross-validated accuracy was 43.0% and the balanced accuracy was 42.1%. These values do not support model stability or clinical utility and show poor discrimination.

As a result, feature-importance values are solely understood as internal descriptive rankings. A verified biomarker effect should not be equated with a comparatively high Gini relevance in a weakly discriminating model.

Given the small cohort size and class imbalance, the goal of this machine learning analysis was not to produce clinically actionable predictive models, but rather to develop hypothesis-supporting feature rankings.

The comparative analysis between operable and inoperable patients yielded several important insights. Inoperable patients exhibited significantly higher NGAL and CEA levels, and lower Vitamin D levels, consistent with a more advanced disease state and potentially compromised immune-nutritional status. While the same top features were retained across groups, subtle shifts in their importance rankings indicate that disease stage may modulate the relevance of specific biomarkers.

Pre-treatment NGAL was higher in inoperable patients (156.22 ± 70.26 ng/mL) than in operable patients (123.49 ± 71.38 ng/mL; Welch p=0.027), according to the operability comparison. It is not appropriate to consider this descriptive/exploratory finding as demonstrating independent prognostic significance.

Because the subgroup estimates were based on the leakage-prone model and limited samples, prior claims of superior Random Forest accuracy in operable versus inoperable patients have been eliminated from the updated interpretation.

Overall, this study confirms that a combination of systemic inflammatory, oncologic, and nutritional markers can be effectively utilized for predictive modeling in cervical cancer. The integration of Random Forest modeling and subgroup stratification reveals both opportunities and limitations in current predictive frameworks. Further studies with larger datasets and more granular outcome definitions are necessary to enhance predictive precision and guide personalized treatment planning.

Although the current data do not establish NGAL as a validated predictor of treatment response, our descriptive findings indicate that NGAL may reflect disease load and biological aggressiveness in cervical cancer. Without clear clinical or imaging evidence, stage-related post-treatment patterns were inconsistent and should not be linked to cytoreduction, nodal sterilization, or therapy-induced inflammation.

Although the current investigation does not show causality, treatment response, or a validated prognostic threshold, the descriptive NGAL patterns across stage and grade remain biologically reasonable. Therefore, rather than being processes supported by this dataset, mechanistic pathways including MMP-9 stabilization, hypoxia-related LCN2 modulation, and neutrophil-mediated inflammation should be considered explanatory hypotheses from the literature.

Overall, NGAL is a dynamic biomarker that merits more research, but this study does not provide a therapeutically useful threshold or monitoring guideline. Future cutoffs should be determined using predetermined ROC methods and verified against objective clinical outcomes in a separate cohort.

There is no suggested clinically useful NGAL threshold or monitoring guideline. Future cutoffs should be externally validated, predetermined, and determined using ROC or another suitable methodology against an objective clinical endpoint.

### 4.1 Study limitations and future directions

The cohort was modest (n = 100), although it comprised all eligible patients with cervical cancer treated at our institution over a two-year period. The stringent exclusion criteria, applied to minimize potentially confounding biochemical influences, together with the limited number of eligible cases in the study setting, restricted the rate at which the dataset could be expanded. Consequently, estimates of machine-learning performance may be unstable and may not be generalizable to other populations or treatment settings.

The model was developed to classify an NGAL-decrease phenotype (post-treatment NGAL < pre-treatment NGAL) rather than an independently verified clinical treatment response. Because the database contained no prespecified radiologic, pathological, or clinical response endpoint, the findings should not be interpreted as evidence of clinical predictive utility. The overlap in biomarker values between groups and the limited cohort size further constrain discrimination and preclude definitive conclusions regarding model performance.

Ongoing patient recruitment will enable extension of the cohort with additional follow-up. Future studies should incorporate larger multicenter datasets, a prespecified independent clinical endpoint, and external validation. These steps will provide more stable estimates of model performance and help determine whether NGAL and conventional biomarkers can contribute to clinically meaningful prediction in cervical cancer.

## 5 Conclusion

This work presents a corrected exploratory Random Forest analysis and describes longitudinal NGAL and conventional biomarker trends in cervical cancer. The model outcome was clearly defined as an NGAL-decrease phenotype rather than treatment response because the provided database lacked an independent objective clinical-response endpoint. To avoid target leakage, all NGAL-derived and post-treatment variables were eliminated.

The leakage-free model did not provide clinical prediction utility due to its weak discrimination (mean 5-fold cross-validated accuracy 43.0%, balanced accuracy 42.1%). NGAL tended to be higher in more advanced broad-stage groups and higher histological grades, and it was descriptively higher in inoperable disease.

Therefore, in this population, NGAL and the related biomarker panel should be regarded as hypothesis-generating. Before any biomarker threshold or machine-learning model can be taken into consideration for clinical decision assistance, future research must have objective, independently defined clinical outcomes, complete FIGO 2018 staging, predetermined statistical plans, and external multicenter validation.

In general, this effort generates hypotheses. Before NGAL or the Random Forest model can be used for clinical decision assistance, a revised analysis utilizing an objective endpoint specified independently of NGAL, complete baseline reporting, FIGO 2018-consistent staging, and external validation in larger multicenter cohorts is necessary.

## Data Availability

The raw data supporting the conclusions of this article will be made available by the authors, without undue reservation.
